# Linking hospital patient records for suspected or established acute coronary syndrome in a complex secondary care system: a proof-of-concept e-registry in National Health Service Scotland

**DOI:** 10.1093/ehjqcco/qcy007

**Published:** 2018-02-16

**Authors:** Iain Findlay, Tamsin Morris, Ruiqi Zhang, Colin McCowan, Sarah Shield, Brian Forbes, Alex McConnachie, Kenneth Mangion, Colin Berry

**Affiliations:** 1 Royal Alexandra Hospital, NHS Greater Glasgow and Clyde Health Board, Corsebar Road, Paisley, UK; 2 AstraZeneca UK, Capability Green, Luton, UK; 3 Robertson Centre for Biostatistics, University of Glasgow, UK; 4 BHF Glasgow Cardiovascular Research Centre, Institute of Cardiovascular and Medical Sciences, University of Glasgow, 126 University Place, Glasgow, Scotland, UK; 5 Golden Jubilee National Hospital, Agamemnon Street, UK

**Keywords:** Acute coronary syndrome, Prognosis, Electronic health records, Registry

## Abstract

**Aims:**

To implement secondary care electronic record linkage for patients hospitalized with suspected or known acute coronary syndrome (ACS) in a complex regional health care system and evaluate this e-Registry in terms of patterns of service delivery and 1-year outcomes.

**Methods and results:**

Existing electronic hospital records were linked to create episodes of care using (i) a patient administration system, (ii) invasive cardiovascular procedure referrals, and (iii) a catheter laboratory record. Data were extracted for admissions (1 October 2013–30 September 2014) with International Classification of Disease (ICD)-10 diagnosis of angina (I200–I209), myocardial infarction (I210–I229), other ischaemic heart disease (I240–I249) or heart failure (I50), linked to other sources to develop a secondary care ACS e-registry and analysed within a Safe Haven. Episodes of care were categorized into care pathways and evaluated in terms of patient characteristics, as well as service delivery metrics and outcomes including mortality. In all, 2327 patients had 2472 episodes of care. Diagnoses were hierarchically classified as ST-elevation myocardial infarction (STEMI) (586, 25.2%), non-ST-elevation myocardial infarction (NSTEMI) (1068, 45.9%), unspecified myocardial infarction (146, 6.3%), unstable angina (527, 22.6%) for the first hospitalization for each patient within the study period. Six care pathways were mapped. Percutaneous coronary intervention rate for STEMI was 80.2% and for NSTEMI 33.1%. Unadjusted all-cause mortality was 9.0% and 3.0% for STEMI and NSTEMI at 30 days, rising to 11.9% and 11.6% at 1 year. Analyses were validated by independent source data verification.

**Conclusion:**

The e-registry has enabled analysis of ACS hospitalizations in a complex health care system with implications for quality improvement and research.

## Introduction

Acute coronary syndrome (ACS) is a leading cause of premature illness and death, and one of the most common reasons for an emergency admission to hospital.^1^^,^^2^ Reducing the public health burden from ACS is a key priority for health care providers and governments.

In the United Kingdom (UK) in 2013/2014, there were 491 647 inpatient episodes attributed to ischaemic heart disease (IHD),^3^ and the number of myocardial infarction (MI) events being recorded in England, Wales, and Northern Ireland has increased (2011/2012—79 433; 2014/2015—83 842).^4^^,^^5^ The Myocardial Ischaemia National Audit Project (MINAP) provides information on national standards of care for patients with heart attack in England, Wales, and Northern Ireland but not in Scotland, where joined-up systems for reporting contemporary secondary care activities and related patient outcomes are lacking. Such information has crucial importance as practice variation in ACS patients has been shown to be associated with mortality,^6–8^ while comparative research across countries might help to improve health systems and prevent deaths.^9^^,^^10^

The National Health Service (NHS) is the sole provider of secondary care services for hospitalized patients with an ACS in the UK. Given the large number of patient episodes and the complexity of this health care system, the process and outcome of individual patients who have been hospitalized with ACS is difficult to describe and uncertain when based on individual hospital records alone.

The project was set up as a Joint Working Project between NHS health boards, including Greater Glasgow and Clyde (NHS GGC) and the Golden Jubilee National Hospital (GJNH) and AstraZeneca UK Ltd. The overall aim of the project was to develop and implement an updatable electronic registry (e-Registry), in a regional network that provides secondary care for patients hospitalized for a known or suspected ACS, with applications for audit, research, and health care improvement. In addition, the project team wished to collaborate and demonstrate that the pharmaceutical industry could work as a trusted partner to the NHS in adding value to health care beyond medicines through shared skills, expertise, and resources to further enhance patient outcomes and service performance.

## Methods

### Setting

The e-Registry was established in the NHS in the West of Scotland. During the study period, NHS GGC provided acute secondary care services through seven hospitals serving a population of approximately 1.2 million. The GJNH is a regional cardiothoracic intervention centre that provides invasive cardiology and cardiothoracic services for this population, amongst others, but is administratively distinct from NHS GGC. These hospitals participate in a Managed Clinical Network to provide strategic health care delivery.

Semi-electronic patient records were implemented across all secondary care clinical and administration systems in NHS GGC and the GJNH by June 2012^11^^,^^12^ enabling capture of key components of ACS care.

### Governance

The project was supported by the National Advisory Committee for Coronary Heart Disease on behalf of the Scottish Government. The Joint Working Project described within this report was approved by hospital management and the Caldicott Guardian for clinical governance in each Health Board.

### Design and methodology

An executable system was developed to identify, extract, and link usual care electronic health records for patients hospitalized with a suspected or known ACS. No new clinical records or manual data entry from paper archives were required. The Community Health Index (CHI) is a 10-digit unique identifier for each person registered with a general practitioner in Scotland and is present on almost all health care encounters. This identifier was used to link the records of patients in the extracted data sets.

**Figure 1:**
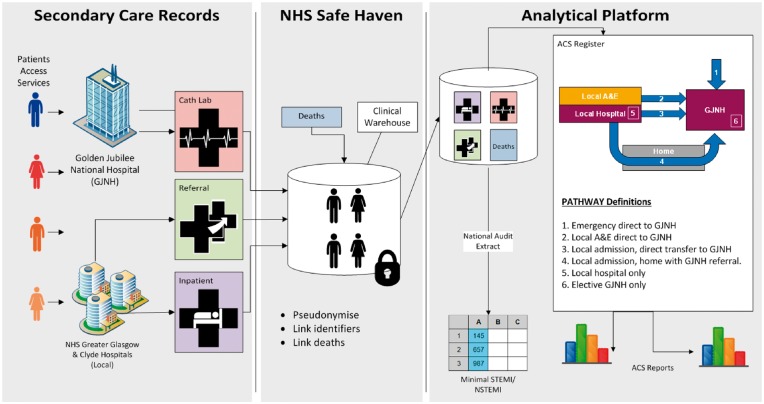
Data sources and linkage.

Hospital patient episodes were used to create episodes of care using (i) the Intersystems TRAKCare Patient Admin System with data extracts based on ICD-10^13^ diagnosis codes, (ii) Scottish Care Information (SCI) Gateway^14^ electronic referrals for invasive cardiovascular procedures, (iii) a bespoke hospital-level patient and catheter laboratory record developed in the GJNH (*Figure 1*).

Data were extracted from these core clinical systems used to manage patients with ACS and deposited within an existing repository for electronic health data (an NHS Safe Haven).^15^ Patients within these data sets were then linked to National Records Scotland (NRS) death certificates where appropriate. The electronic records were pseudonymised within the Safe Haven before being securely transferred to the analysts who were employed by AstraZeneca UK Ltd under a data sharing agreement signed by all parties. Statistical analysis was supported by a co-funded PhD from the University of Glasgow.

The ACS diagnoses were based on the discharge summary recorded by the attending clinician(s) in usual care health records captured in the clinical systems. In the local hospital patient administration system (TRAKCare), the diagnoses are subsequently coded per the International Classification of Disease (ICD)-10 and in the invasive centre (IC) the discharge diagnosis is recorded in a standardized text format. An algorithm was developed to assign the most appropriate diagnosis to an episode of care. Where a patient had multiple diagnoses in a linked episode of care from different clinical settings or systems, the diagnosis on discharge from the IC was taken as the most accurate representation. Where a patient did not attend the IC as part of their care pathway, the ICD-10 diagnosis from the local hospital was used. As well as this a hierarchy of diagnoses was used to ensure that the most severe or specific final diagnosis was acknowledged, with ST-elevation myocardial infarction (STEMI) at the top of the hierarchy (see Supplementary material online).

Data were extracted from TRAKCare for all admissions (1 October 2013–30 September 2014) with an ICD-10 diagnosis of angina (I200–I209), myocardial infarction (I210–I229), other IHD (I240–I249), or heart failure (I50) to ensure complete capture of suspected ACS events. This was linked to referrals for invasive cardiovascular procedures made through the SCI-Gateway system, cardiac interventions performed in the IC of the GJNH and all-cause mortality data from NRS. This linked data set was then analysed to look at diagnoses and patient characteristics, and to identify episodes of care, which were then categorized into distinct clinical care pathways. Those with a final diagnosis of ACS were isolated for analysis on referrals for invasive cardiovascular procedures, treatment durations, service delivery metrics and outcomes, including mortality.

After the classification of patients and pathways, data on deprivation status of patients was provided by the NHS Safe Haven identified based on the postcode of the patient’s home address and measured using quintiles of the Scottish Index of Multiple Deprivation (SIMD) 2012 measure.^16^^,^^17^ Quintile 1 represents the highest level of deprivation with Q5 representing the least deprived. The top 20% most deprived data zones in Scotland are in the first quintile, with the distribution of Glasgow City’s data zones being 49%, 19%, 13%, 10.5%, 8.5% (Q1–Q5).^16^

### Pre-specified health care outcomes

The pre-specified primary outcomes were 30 day and 1 year all-cause mortality. The receipt of cardiac interventions, duration of hospital stay and pathways of care were the pre-specified secondary outcomes.

### Data validation

A quality assurance procedure was conducted by the NHS in Glasgow to assess the robustness of the data extraction process and the accuracy of the outputs from the analysis programme, as compared with source clinical data assessed and verified by independent, trained observers. The review focused on (i) a quality assessment of 200 individual patient episodes, taken from a randomly selected calendar month (30 days) of the e-Registry with all consecutive episodes included, and (ii) an assessment of the causes of death of 44 patients.

The observers accessed usual care health records using TRAKCare and other executable systems including Clinical Portal^18^ and SCI-STORE^19^ that are used at the point of care in hospital.

A team of five research nurses with a cardiovascular background employed by the NHS in Glasgow were tasked with assessing source clinical data against the data from the e-registry to confirm or query the patient episodes, including dates and the primary and secondary causes of these events. In addition, two cardiologists (K.M., C.B.) assessed any queries that were raised by the research nurses.

The reviewers focused on 4 aspects of the analysis programme; care pathway assignment, diagnosis assignment, assessment of invasive procedures received and assessment of mortality.

### Statistical analyses

As this project involved exploratory health services delivery research, a power calculation was not performed. Categorical variables are expressed as number and percentage of patients. Most continuous variables followed a normal distribution and are therefore presented as means together with standard deviation. Those variables that did not follow a normal distribution are presented as medians with interquartile range. Differences in continuous variables between groups were assessed by the t-test or Mann-Whitney test as appropriate. Differences in categorical variables between groups were assessed using the Fisher’s test. Cox proportional hazards regressions were used to evaluate the effect of risk factors and intervention procedures on all-cause mortality for ST-segment elevation myocardial infarction (STEMI) and non-STEMI (NSTEMI) patients. Multivariable logistic or zero-inflated negative binomial models were performed to evaluate differences in service delivery. Survival analyses will be performed using the Kaplan-Meier method. Analyses were conducted using SAS Enterprise Guide (v5.1).

## Results

### Demographics and clinical characteristics

Between 1 October 2013 and 30 September 2014 (12 months), 2327 unique patients had 2472 distinct episodes of care across 7 acute hospitals and one regional cardiothoracic centre. Of these patients, the final diagnosis was STEMI in 586 (25.2%) episodes, NSTEMI in 1068 (45.9%), unspecified MI in 146 (6.3%), and unstable angina (UA) in 527 (22.6%) for their first hospitalization within the study period. ST-elevation myocardial infarction patients were generally younger with a higher proportion of males than the other diagnoses and relatively more deprived than NSTEMI (77.6% in SIMD Q1–3 vs. 73.6%) (see *Table 1*).

**Table 1:** Demographic characteristics by final diagnosis

	All (*n* = 2327)	STEMI (*n* = 586)	NSTEMI (*n* = 1068)	Unspecified MI (*n* = 146)	Unstable angina (*n* = 527)
Age at admission (years)	67.5 (13.9)	62.8 (14.2)	68.4 (13.4)	77.0 (11.6)	68.3 (13.2)
Gender					
Female	989 (42.5%)	182 (31.1%)	458 (42.9%)	70 (47.9%)	279 (52.9%)
Male	1338 (57.5%)	404 (68.9%)	610 (57.1%)	76 (52.1%)	248 (47.1%)
Ethnicity					
White	1636 (70.4%)	404 (69.2%)	650 (60.9%)	130 (89.0%)	452 (85.8%)
Other	48 (2.1%)	8 (1.4%)	23 (2.2%)	4 (2.7%)	13 (2.5%)
Unknown	643 (27.6%)	174 (29.5%)	395 (37.0%)	12 (8.2%)	62 (11.8%)
Missing	2	2	0	0	0
SIMD quintile					
Q1 (most deprived)	906 (43.6%)	214 (41.2%)	407 (43.4%)	63 (44.7%)	222 (46.3%)
Q2	372 (17.9%)	95 (18.3%)	167 (17.8%)	24 (17.0%)	86 (18.0%)
Q3	293 (14.1%)	94 (18.1%)	116 (12.4%)	25 (17.7%)	58 (12.1%)
Q4	245 (11.8%)	50 (9.6%)	113 (12.0%)	15 (10.6%)	67 (14.0%)
Q5 (least deprived)	262 (12.6%)	67 (12.9%)	135 (14.4%)	14 (9.9%)	46 (9.6%)
Missing	249	66	130	5	48
Pathway					
Emergency direct to IC	333 (14.3%)	304 (51.9%)	29 (2.7%)	0 (0.0%)	0 (0.0%)
Local A&E to IC	155 (6.7%)	148 (25.3%)	7 (0.7%)	0 (0.0%)	0 (0.0%)
Acute invasive	492 (21.1%)	57 (9.7%)	426 (39.9%)	2 (1.4%)	7 (1.3%)
Elective invasive	208 (8.9%)	5 (0.9%)	198 (18.5%)	0 (0.0%)	5 (0.9%)
Local hospital only	1081 (46.5%)	68 (11.6%)	364 (34.1%)	144 (98.6%)	505 (95.8%)
Elective direct to IC	58 (2.5%)	4 (0.7%)	44 (4.1%)	0 (0.0%)	10 (1.9%)

Data are mean (SD) or number (%).

SIMD, Scottish Index of Multiple Deprivation; IC, Invasive Centre. For first admission in study period.

**Figure 2:**
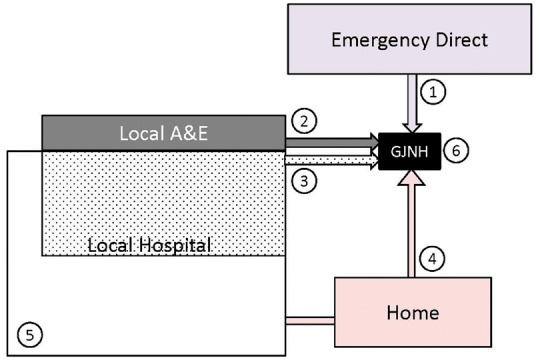
All acute coronary syndrome patients (1st hospitalization in timeframe) by care pathway. Note sizes are proportional to percentage of analysis population. Pathway definitions: 1—Emergency direct admission to invasive centre [Golden Jubilee National Hospital (GJNH)]; 2—Local A&E in regional hospital followed by direct transfer to invasive centre; 3—Admission to local hospital followed by inter-hospital transfer to the invasive centre (acute invasive); 4—Admission to local hospital followed by referral to the invasive centre on an urgent outpatient basis (elective invasive); 5—Local hospital only (no referral for invasive management); 6—Elective direct to the invasive centre (no local hospital referral).

Six distinct treatment pathways were identified (*Figure 2*). The treatment pathway was mapped for each episode of care giving 53% of patients admitted to the IC; 14% directly as emergency episodes; 3% directly on an elective basis; 7% via local A&E; 21% after an inpatient stay in a local hospital for acute invasive care; and 9% discharged home from the local hospital for elective invasive care at a later date. The other 47% stayed within the local hospital only, either managed conservatively with no following invasive treatment or dying in hospital (*Table 1*).

As expected and dictated by local protocol, STEMI patients tend to be admitted to the IC directly or by transfer from the local A&E for immediate invasive management while NSTEMI patients tend to be transferred to the IC after admission to a local hospital (*Table 1*).

Most records of clinical characteristics and cardiovascular risk factors came from the IC or electronic referrals for invasive cardiovascular procedures. These are provided in *Tables 2 and 3* for the STEMI and NSTEMI patients undergoing invasive management, respectively. Patients not undergoing invasive management did not have these data recorded as they did not enter a care pathway with systems that provided this information. Where information was available, patients with unstable angina/NSTEMI tended to have more concomitant diseases including hypertension, hypercholesterolaemia, diabetes, a family history of CHD and histories of MI, PCI, CABG and symptomatic peripheral vascular disease than patients with STEMI. However, patients with STEMI were more likely to be current smokers.

**Table 2:** **Clinical characteristics for** ST-elevation myocardial infarction patients **undergoing invasive treatment by intervention**

	Angiogram	PCI after angiogram	
	Yes (*n* = 504)	No (*n* = 36)	Yes (*n* = 468)
Risk factors			
Age at admission (years)	60.9 (13.1)	59.0 (16.2)	61.0 (12.8)
Gender			
Female	142 (28.2%)	6 (16.7%)	136 (29.1%)
Male	362 (71.8%)	30 (83.3%)	332 (70.9%)
SIMD quintile			
Q1 (most deprived)	184 (36.5%)	10 (27.8%)	174 (37.2%)
Q2	83 (16.5%)	3 (8.3%)	80 (17.1%)
Q3	82 (16.3%)	6 (16.7%)	76 (16.2%)
Q4	45 (8.9%)	4 (11.1%)	41 (8.8%)
Q5 (least deprived)	56 (11.1%)	5 (13.9%)	51 (10.9%)
Hypertension	190 (37.7%)	16 (44.4%)	174 (37.2%)
Hypercholesterolaemia	162 (32.1%)	12 (33.3%)	150 (32.1%)
Diabetes	36 (7.1%)	3 (8.3%)	33 (7.1%)
Smoking status			
Current	250 (49.6%)	11 (30.6%)	239 (51.1%)
Ex	101 (20.0%)	14 (38.9%)	87 (18.6%)
Never	119 (23.6%)	6 (16.7%)	113 (24.1%)
Family history of CHD	192 (38.1%)	13 (36.1%)	179 (38.2%)
Medical history			
Previous PCI	54 (10.7%)	2 (5.6%)	52 (11.1%)
Previous cardiac surgery			
CABG	10 (2.0%)	1 (2.8%)	9 (1.9%)
CABG; valve	2 (0.4%)	0 (0.0%)	2 (0.4%)
None/missing	490 (97.2%)	35 (97.2%)	455 (97.2%)
Other cardiac	1 (0.2%)	0 (0.0%)	1 (0.2%)
Valve	1 (0.2%)	0 (0.0%)	1 (0.2%)
Previous MI	73 (14.5%)	6 (16.7%)	67 (14.3%)

Data are mean (SD) or number (%) out of group total.

CABG, coronary artery bypass grafting surgery; CHD, coronary heart disease; PCI, percutaneous coronary intervention; SIMD, Scottish Index of Multiple Deprivation.

**Table 3:** **Clinical characteristics for non-**ST-elevation myocardial infarction patients **undergoing invasive treatment by intervention**

	Angiogram	PCI after angiogram	
	Yes (*n* = 678)	No (*n* = 334)	Yes (*n* = 344)
Risk factors			
Age at admission (years)	63.7 (11.7)	63.8 (11.7)	63.7 (11.7)
Gender			
Female	253 (37.3%)	133 (39.8%)	120 (34.9%)
Male	425 (62.7%)	201 (60.2%)	224 (65.1%)
SIMD quintile			
Q1 (most deprived)	263 (38.8%)	140 (41.9%)	123 (35.8%)
Q2	104 (15.3%)	47 (14.1%)	57 (16.6%)
Q3	76 (11.2%)	39 (11.7%)	37 (10.8%)
Q4	64 (9.4%)	31 (9.3%)	33 (9.6%)
Q5 (least deprived)	85 (12.5%)	33 (9.9%)	52 (15.1%)
Hypertension	363 (53.5%)	179 (53.6%)	184 (53.5%)
Hypercholesterolaemia	297 (43.8%)	146 (43.7%)	151 (43.9%)
Diabetes	67 (9.9%)	28 (8.4%)	39 (11.3%)
Smoking status			
Current	221 (32.6%)	98 (29.3%)	123 (35.8%)
Ex	166 (24.5%)	82 (24.6%)	84 (24.4%)
Never	222 (32.7%)	119 (35.6%)	103 (29.9%)
Family history of CHD	299 (44.1%)	144 (43.1%)	155 (45.1%)
BMI (kg/m^2^)	29.0 (5.7)	28.8 (5.9)	29.2 (5.6)
Missing	129	59	70
GRACE Score	134.9 (37.6)	135.2 (39.2)	134.6 (36.0)
Missing	89	38	51
Medical history			
Previous PCI	101 (14.9%)	47 (14.1%)	54 (15.7%)
Previous Cardiac Surgery			
CABG	41 (6.0%)	18 (5.4%)	23 (6.7%)
CABG; Valve	1 (0.1%)	0 (0.0%)	1 (0.3%)
Congenital cardiac	1 (0.1%)	0 (0.0%)	1 (0.3%)
None/missing	628 (92.6%)	313 (93.7%)	315 (91.6%)
Other cardiac	5 (0.7%)	2 (0.6%)	3 (0.9%)
Valve	2 (0.3%)	1 (0.3%)	1 (0.3%)
Previous MI	173 (25.5%)	90 (26.9%)	83 (24.1%)

Data are mean (SD) or number (%) out of group total. GRACE scores are recorded at time of referral.

BMI, body mass index; CABG, coronary artery bypass grafting surgery; CHD, coronary heart disease; PCI, percutaneous coronary intervention; SIMD, Scottish Index of Multiple Deprivation.

### Service Delivery

Intervention rates and the mean number of days to invasive procedure varied depending on care pathway. The mean total duration of hospital stay for all patients with a diagnosis of STEMI was lower than for all patients with a diagnosis of NSTEMI (5.5, 95% CI 4.8–6.2 days vs. 7.5, 95% CI 6.8–8.1 days; *P* < 0.0001). These durations include both those in invasive and non-invasive pathways. Among all patients with STEMI (including those who remained in the local hospital), 470 (80.2%) underwent reperfusion therapy by percutaneous coronary intervention (PCI), while NSTEMI (33.1%) and UA (1.9%) patients underwent revascularization by PCI significantly less frequently. For patients managed invasively, STEMI bed days after intervention were higher than for NSTEMI (1.4, 95% CI 1.3–1.5 days vs. 1.1, 95% CI 1.0–1.2 days; *P* < 0.0001) whereas NSTEMI patients spent longer in hospital prior to intervention (0.7, 95% CI 0.4–1.0 days vs. 4.4, 95% CI 4.0–4.8 days; *P* < 0.0001). This finding is related to the differences observed in the repatriation of patients to local hospitals depending on a STEMI or NSTEMI diagnosis. 85.3% of STEMI patients treated in the IC were repatriated following treatment as compared to 19.9% of NSTEMI patients, who tended to be discharged home directly from the IC. Other characteristics of service delivery by diagnosis (all pathways combined) are provided in the Supplementary material online, *Table S8*.

Of the episodes of care where a STEMI patient accessed the IC for intended invasive treatment, 90.7% of episodes involved the patient receiving PCI. The median door-to-balloon (time from arrival at IC to receipt of PCI) (DTB) and call-to-balloon (time from call to emergency services to receipt of PCI) (CTB) times were 22 and 95 min, respectively for STEMI patients (*Table 4*). This compares favourably to the median DTB and CTB times presented in the most recent MINAP Annual Report^5^ for England which were 41 min and 115 min, respectively. Myocardial Ischaemia National Audit Project also measures the proportions of STEMI patients who receive their PCI against different time metrics. Times to receipt of PCI for patients treated in Glasgow again compare favourably to England for all reported metrics; proportion of patients receiving PCI within (i) 90 min of arriving at IC (Glasgow 98.5% vs. England 88.9%), (ii) 150 min of call for help whether direct or transfer (85.9% vs. 82.3%), (iii) 150 min of call for help with direct admission (94.8% vs. 82.8%), (iv) 150 min of call for help admitted by transfer (60.9% vs. 50.5%), and (v) 120 min of call for help with direct admission (87.5% vs. 53.9%).

**Table 4:** **Service delivery by pathway for** ST-elevation myocardial infarction **and non-**ST-elevation myocardial infarction **for those intended for invasive treatment**

	All	Emergency direct to IC	Local A&E to IC	Acute invasive	Elective invasive	Elective direct to IC
STEMI (*n*)	518	304	148	57	5	4
Receipt of coronary angiography						
Yes	504 (97.3%)	301 (99.0%)	146 (98.6%)	49 (86.0%)	5 (100.0%)	3 (75.0%)
No	14 (2.7%)	3 (1.0%)	2 (1.4%)	8 (14.0%)	0 (0.0%)	1 (25.0%)
Duration from admission to angiography (days)	0 [0, 0]	0 [0, 0]	0 [0, 0]	1 [0, 3]	18 [18, 21]	0 [0, 0]
Receipt of PCI						
Yes	470 (90.7%)	285 (93.8%)	138 (93.2%)	40 (70.2%)	3 (60.0%)	4 (100.0%)
No	48 (9.3%)	19 (6.3%)	10 (6.8%)	17 (29.8%)	2 (40.0%)	0 (0.0%)
Duration from admission to PCI (days)	0 [0, 0]	0 [0, 0]	0 [0, 0]	1 [0, 3]	21 [11, 23]	0 [0, 0]
Call-to-balloon (min)	95 [83, 116]	91 [81, 104]	132 [99, 191]	76 [74, 108]	NR	NR
*n*	325	232	87	5	NR	NR
Door-to-balloon (min)	22 [18, 27]	21 [18, 27]	22 [18, 29]	25 [14, 27]	NR	NR
*n*	400	263	126	10	NR	NR
NSTEMI (*n*)	704	29	7	426	198	44
Receipt of coronary angiography						
Yes	678 (96.3%)	27 (93.1%)	7 (100.0%)	408 (95.8%)	198 (100.0%)	38 (86.4%)
No	26 (3.7%)	2 (6.9%)	0 (0.0%)	18 (4.2%)	0 (0.0%)	6 (13.6%)
Duration from admission to angiography (days)	5 [2, 14]	0 [0, 0]	0 [0, 0]	4 [2, 6]	23 [16, 29]	0 [0, 0]
Receipt of PCI						
Yes	354 (50.3%)	18 (62.1%)	6 (85.7%)	233 (54.7%)	72 (36.4%)	25 (56.8%)
No	350 (49.7%)	11 (37.9%)	1 (14.3%)	193 (45.3%)	126 (63.6%)	19 (43.2%)
Duration from admission to PCI (days)	4 [1, 9]	0 [0, 0]	0 [0, 0]	4 [2, 6]	23 [15, 27]	0 [0, 0]

The recommended call-to-balloon time is within 150 min and door-to-balloon time within 90 min for STEMI.^5^^,^^20^ The recommended duration from admission to angiography for NSTEMI is within 72 h (3 days).^21^ Data are number (%) or median [IQR]. Where duration from admission to angiography/PCI is 0 days, this means the procedure happened on the same day as admission.

NR, Not relevant.

For the 63.5% of NSTEMI patients who were referred to the IC for intended invasive treatment, 96.3% underwent angiography, but only 50.3% then went on to have PCI. In comparison, the MINAP report indicates that 79.0% of patients in England were referred for angiography during their admission for NSTEMI in 2014–2015. The proportion of NSTEMI patients seen by a cardiologist for Glasgow and England were also able to be compared (93.3% vs. 95.1%). Time to angiography for NSTEMI patients differed by pathway of care (*Table 4*). For NSTEMI patients referred to the IC via the acute invasive pathway, median time to angiography was 4 days with 25% receiving angiography within 2 days and 75% receiving angiography within 6 days. For those who accessed the IC via the elective invasive pathway median time to angiography was 23 days (IQR 16–29 days).

### Associations with all-cause mortality and service delivery

The unadjusted all-cause mortality rate at 30 days was 9.0% in all STEMI patients and 3.0% in all NSTEMI patients rising to 11.9% in STEMI and 11.6% in NSTEMI at 1 year. Age-, gender-, and pathway-adjusted 30-day mortality in STEMI was significantly higher than that in NSTEMI (HR 4.63, 95% CI 2.7–7.92) and remains higher at 1 year (HR 1.72, 95% CI 1.16–2.53).

**Figure 3:**
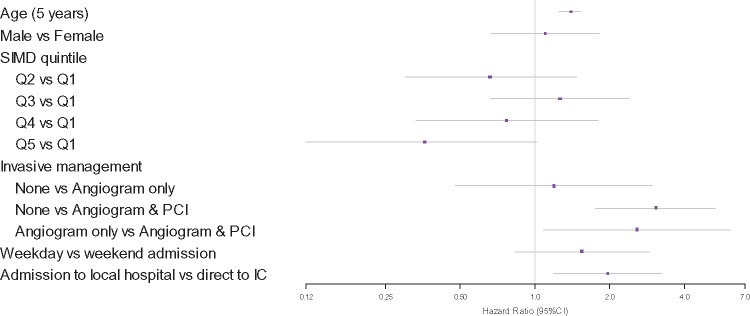
Associations with all-cause mortality at 1 year for ST-elevation myocardial infarction patients. SIMD, Scottish Index of Multiple Deprivation. Q1 represents the highest level of deprivation.

**Figure 4:**
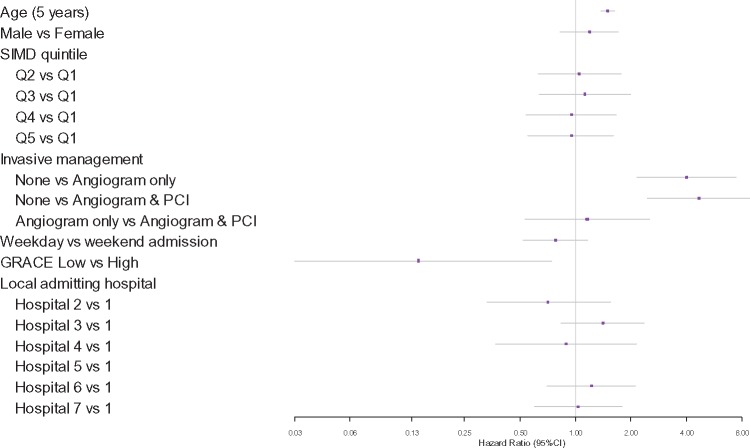
Associations with all-cause mortality at 1 year for non-ST-segment elevation myocardial infarction patients. SIMD, Scottish Index of Multiple Deprivation. Q1 represents the highest level of deprivation.

Age-adjusted associations of all-cause death in STEMI and NSTEMI patients at 30 days (*Table 5*) and 1 year (*Figure 3* and *4*) were assessed. Compared to conservative non-invasive treatment, the receipt of angiography alone is not associated with increased survival at 30 days or 1 year in STEMI patients but the receipt of PCI was associated with higher survival (30 days: HR 3.07, 95% CI 1.61–5.85, 1 year: HR 3.06, 95% CI 1.75–5.34). Initial presentation in a local hospital compared to being admitted to the IC directly is associated with higher mortality for STEMI patients (30 days: HR 1.86, 95% CI 1.04–3.32; *P* = 0.036, 1 year: HR 1.96, 95% CI 1.19–3.24; *P* = 0.009) but the association disappears after adjusting for the rate of PCI performed. For NSTEMI patients, the receipt of angiography alone (30 days: HR 6.95, 95% CI 1.55–31.17, 1 year: HR 4.03, 95% CI 2.17–7.48) and angiography with follow-on PCI (30 days: HR 3.66, 95% CI 1.18–11.40, 1 year: HR 4.68, 95% CI 2.48–8.83) are both associated with higher survival, with no statistically significant difference between the two. GRACE score was predictive of all-cause mortality at 1-year for NSTEMI (*P* = 0.021) but not at 30 days (*P* = 0.402). There was no difference in 30-day (*P* = 0.238) or 1 year (*P* = 0.676) mortality between local admitting hospitals.

**Table 5:** **Associations with 30-day mortality in all** ST-elevation myocardial infarction **and non-**ST-elevation myocardial infarction **patients**

30-day mortality and associations	STEMI		NSTEMI	
	Hazard ratio (95% CI)	*P*-value	Hazard ratio (95% CI)	*P*-value
Age (5 year)	1.31 (1.18, 1.46)	<0.0001	1.44 (1.23, 1.69)	<0.0001
Male vs. female	1.05 (0.59, 1.87)	0.8717	1.82 (0.88, 3.77)	0.1056
SIMD quintile		0.1148		0.7562
Q2 vs. Q1	0.61 (0.22, 1.64)		1.20 (0.45, 3.20)	
Q3 vs. Q1	1.60 (0.77, 3.29)		1.08 (0.35, 3.36)	
Q4 vs. Q1	1.04 (0.41, 2.67)		1.46 (0.55, 3.89)	
Q5 vs. Q1	0.27 (0.06, 1.19)		0.57 (0.16, 2.03)	
Admission to local hospital vs. direct admission to IC (all patients)	1.86 (1.04, 3.32)	0.0363	NA	
Admission to local hospital vs. direct admission to IC (patients intended for invasive management)	0.70 (0.21, 2.28)	0.5488	NA	
GRACE (low vs. high)	NA		0.31 (0.02, 4.86)	0.4019
Invasive management		0.0012		0.0082
None vs. angiogram only	1.10 (0.40, 3.05)		6.95 (1.55, 31.17)	
None vs. angiogram and PCI	3.07 (1.61, 5.85)		3.66 (1.18, 11.40)	
Angiogram only vs. angiogram and PCI	2.79 (1.08, 7.25)		0.53 (0.10, 2.88)	
Local admitting hospital	NA			0.2376
Hospital 2 vs. 1			0.21 (0.03, 1.58)	
Hospital 3 vs. 1			0.89 (0.37, 2.16)	
Hospital 4 vs. 1			1.04 (0.30, 3.66)	
Hospital 5 vs. 1			—	
Hospital 6 vs. 1			0.39 (0.11, 1.38)	
Hospital 7 vs. 1			0.20 (0.05, 0.89)	

GRACE scores are recorded at time of referral. GRACE score: low (≤140), high (>140).

IC, Intervention Centre; PC, percutaneous coronary intervention; SIMD, Scottish Index of Multiple Deprivation; Q1, most deprived; Q5, least deprived.

**Figure 5:**
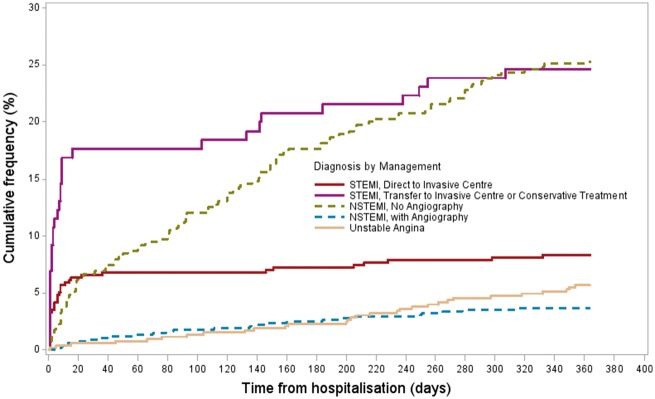
The Kaplan–Meier plot for all-cause mortality by diagnosis and management.

All-cause mortality by diagnosis and management is shown in *Figure 5*. Age-adjusted associations of service delivery are shown in *Table 6*. If referred for intervention, NSTEMI patients with high GRACE scores tend to be fast-tracked through an acute invasive pathway (*P* < 0.001) but did not differ in the rate of angiography or PCI compared to those referred with low GRACE scores. Males experiencing NSTEMI were more likely to undergo angiography (OR 1.40, 95% CI 1.06–1.87) and PCI (OR 1.41, 95% CI 1.08–1.85) than females but gender was not apparently associated with whether a patient was referred to the intervention centre (OR 1.31, 95% CI 0.97–1.77) or whether a referral was acute invasive or elective invasive (OR 1.14, 95% CI 0.80–1.64). However, crude referral rates for females and males were 55.1% and 69.0%, respectively, suggesting that there is a numerical difference between gender. In those referred, female patients tended to have higher GRACE scores with 48.2% having a GRACE score >140 vs. 40.9% in males. Male gender was not associated with increased 30-day or 1-year mortality. Males were more likely than females to be diagnosed with NSTEMI vs. unstable angina (OR 1.50, 95% CI 1.22–1.85). Younger patients were more likely to be referred for angiography and PCI, but if referred, older patients tended to be fast-tracked through an acute invasive pathway, likely to be in part explained by the effect of age on the GRACE score. There was a large proportion (46%) of NSTEMI patients referred for angiography that did not receive follow-on PCI. Referral and angiography rates for NSTEMI patients differed significantly depending on the local admitting hospital (*P* = 0.03 and *P* = 0.011, respectively). Local admitting hospital also had an association with whether a patient was referred to the IC via an acute or elective invasive pathway (*P* < 0.0001) as well as the time from local hospital referral to receipt of PCI (*P* < 0.0001). Despite the differences in angiography referrals, there was ultimately no significant association between local admitting hospital and receipt of PCI (*Table 6*).

**Table 6:** **Associations with service delivery in all** ST-elevation myocardial infarction **and non-**ST-elevation myocardial infarction **patients**

Service delivery and associations	STEMI		NSTEMI	
	Odds ratio/estimate (95% CI)	*P*-value	Odds ratio/estimate (95% CI)	*P*-value
Angiography (%)	86.0		63.5	
Age (5 year)	0.68 (0.61, 0.75)	<0.0001	0.64 (0.60, 0.69)	<0.0001
Male vs. Female	1.43 (0.85, 2.41)	0.1742	1.40 (1.06, 1.87)	0.0198
SIMD quintile		0.4777		0.6751
Q2 vs. Q1	1.23 (0.56, 2.68)		0.90 (0.59, 1.38)	
Q3 vs. Q1	1.48 (0.68, 3.24)		1.09 (0.67, 1.79)	
Q4 vs. Q1	2.62 (0.90, 7.60)		0.92 (0.57, 1.49)	
Q5 vs. Q1	1.19 (0.63, 2.68)		1.31 (0.83, 2.09)	
Admission to local hospital vs. direct admission to IC	0.01 (0.01, 0.03)	<0.0001	NA	
GRACE (low vs. high)	NA		2.36 (0.71, 7.83)	0.1590
Local admitting hospital	NA			0.0111
Hospital 2 vs. 1			0.52 (0.30, 0.90)	
Hospital 3 vs. 1			1.00 (0.65, 1.55)	
Hospital 4 vs. 1			0.80 (0.41, 1.55)	
Hospital 5 vs. 1			—	
Hospital 6 vs. 1			0.45 (0.29, 0.71)	
Hospital 7 vs. 1			0.75 (0.48, 1.16)	
Percutaneous coronary intervention (%)	80.2		33.1	
Age (5 year)	0.79 (0.73, 0.86)	<0.0001	0.82 (0.78, 0.86)	<0.0001
Male vs. female	1.13 (0.72, 1.78)	0.5949	1.41 (1.08, 1.85)	0.0132
SIMD quintile		0.8286		0.1363
Q2 vs. Q1	1.30 (0.66, 2.53)		1.29 (0.87, 1.92)	
Q3 vs. Q1	1.12 (0.59, 2.12)		1.17 (0.74, 1.84)	
Q4 vs. Q1	1.44 (0.63, 3.30)		1.19 (0.74, 1.90)	
Q5 vs. Q1	0.91 (0.46, 1.81)		1.75 (1.15, 2.68)	
Admission to local hospital vs. direct admission to IC	0.04 (0.02, 0.07)	<0.0001	NA	
GRACE (low vs. high)	NA		0.83 (0.55, 1.24)	0.3566
Local admitting hospital	NA			0.1059
Hospital 2 vs. 1			0.80 (0.46, 1.38)	
Hospital 3 vs. 1			1.13 (0.76, 1.70)	
Hospital 4 vs. 1			0.93 (0.50, 1.74)	
Hospital 5 vs. 1			—	
Hospital 6 vs. 1			0.61 (0.38, 0.97)	
Hospital 7 vs. 1			1.29 (0.86, 1.92)	
Referral to intervention centre (invasive transfer vs. local hospital only) (%)	47.7		63.2	
Age (5 year)	0.66 (0.56, 0.77)	<0.0001	0.64 (0.59, 0.68)	<0.0001
Male vs. female	1.84 (0.76, 4.48)	0.1774	1.31 (0.97, 1.77)	0.0751
SIMD quintile		0.0843		0.6356
Q2 vs. Q1	1.57 (0.42, 5.91)		0.96 (0.62, 1.50)	
Q3 vs. Q1	1.17 (0.30, 4.59)		1.15 (0.69, 1.91)	
Q4 vs. Q1	5.14 (1.23, 21.42)		0.98 (0.60, 1.61)	
Q5 vs. Q1	0.39 (0.09, 1.66)		1.41 (0.87, 2.29)	
Local admitting hospital	NA			0.0301
Hospital 2 vs. 1			0.61 (0.35, 1.07)	
Hospital 3 vs. 1			0.98 (0.63, 1.52)	
Hospital 4 vs. 1			0.81 (0.41, 1.58)	
Hospital 5 vs. 1			—	
Hospital 6 vs. 1			0.46 (0.29, 0.73)	
Hospital 7 vs. 1			0.71 (0.46, 1.11)	
Acute vs. elective invasive transfer (%)	91.9		68.3	
Age (5 year)	1.27 (0.88, 1.82)	0.1973	1.25 (1.16, 1.35)	<0.0001
Male vs. female	—	0.9566	1.14 (0.80, 1.64)	0.4740
SIMD quintile		0.5882		0.1113
Q2 vs. Q1	—		0.54 (0.32, 0.90)	
Q3 vs. Q1	0.36 (0.02, 7.72)		0.97 (0.54, 1.76)	
Q4 vs. Q1	0.20 (0.01, 5.81)		1.17 (0.60, 2.30)	
Q5 vs. Q1	0.10 (0.01, 1.51)		0.72 (0.41, 1.26)	
GRACE (low vs. high)	NA		0.07 (0.04, 0.13)	<0.0001
Local admitting hospital	NA			<0.0001
Hospital 2 vs. 1			4.50 (1.52, 13.32)	
Hospital 3 vs. 1			0.60 (0.36, 0.98)	
Hospital 4 vs. 1			0.70 (0.33, 1.48)	
Hospital 5 vs. 1			—	
Hospital 6 vs. 1			0.33 (0.19, 0.58)	
Hospital 7 vs. 1			1.00 (0.58, 1.70)	
Time from admission to PCI (days)				
Age (5 year)	−0.23 (−0.52, 0.05)	0.1050	−0.12 (−0.18, −0.06)	<0.0001
Weekday vs. weekend admission	1.08 (−0.36, 2.52)	0.1426	0.03 (−0.29, 0.34)	0.8610
Admission to local hospital vs. direct admission to IC	4.78 (4.01, 5.55)	<0.0001	NA	
Local admitting hospital	NA			<0.0001
Hospital 2 vs. 1			−0.43 (−0.91, 0.04)	
Hospital 3 vs. 1			0.63 (0.29, 0.97)	
Hospital 4 vs. 1			0.15 (−0.36, 0.67)	
Hospital 5 vs. 1			—	
Hospital 6 vs. 1			0.42 (0.02, 0.82)	
Hospital 7 vs. 1			−0.09 (−0.41, 0.23)	

GRACE scores are recorded at time of referral. GRACE score: low (≤140) high (>140).

IC, Intervention Centre; PCI, percutaneous coronary intervention; SIMD, Scottish Index of Multiple Deprivation; Q1, Most deprived; Q5, Least deprived.

A patient’s deprivation status (as measured by the SIMD) was not seen to be associated with either mortality at 30 days or 1 year, or the pathway of care for STEMI or NSTEMI patients including delivery of angiography or PCI.

### Outcome of data validation

Following review of source clinical data, pathway assignment was confirmed to be correct in all [*n* = 200 (100%)] of the patient episodes. The diagnosis assignment was confirmed in 199 (99%) and one subject had missing data. The occurrence of invasive coronary angiography, PCI and the dates of these procedures, were confirmed for all subjects [*n* = 200 (100%)].

Forty-four individual patient records were included in the verification of mortality assignment. All deaths were confirmed. The cause of death was confirmed to be correct in 100% of the deceased patients with available records [*n* = 36 (82%)]. Verification of cause of death was not possible in eight subjects (18%) in whom primary care records could not be accessed. The date of death was confirmed correct in all (100%) subjects.

## Discussion

The primary objective of this proof-of-concept project was to implement a contemporary secondary care e-Registry for patients hospitalized with suspected or established ACS, utilizing only electronic records collected as part of usual clinical care in a complex regional health care system; and to be able to produce clinically meaningful analyses from these data without the need for additional manual data collection. The pilot has demonstrated that implementation of an e-registry is possible with the main outcomes being (i) longitudinal follow-up of patient episodes for all-cause and cause-specific mortality, (ii) a new system that has potential applications for quality and health care improvement, service evaluation and electronic health record-enabled research, and (iii) a system that could enable reporting for national audit.

The analyses performed on the data suggest possible variation in standards of care and service delivery for patients experiencing STEMI and NSTEMI, including differing service provision depending on the local admitting hospital. As has previously been demonstrated in other studies, whether a patient receives invasive management or not is clearly associated with patient outcomes including mortality. The 2015 ESC Guidelines for the management of ACS in patients presenting without persistent ST-segment elevation^21^ recommend that patients with at least 1 intermediate risk factor should receive angiography within 72 h (3 days) of hospital admission. At the time of data extraction for this analysis the acute invasive pathway was the most likely route through which intermediate to high-risk NSTEMI patients would access the IC. However, in this pathway less than 50% of NSTEMI patients achieved this recommendation. Based partly on the evidence provided by the e-registry a ‘Direct NSTEMI’ service has recently been implemented whereby patients with high-risk characteristics would be directly transferred by the Ambulance Service to the IC, rather than the usual admission pathway to the nearest local hospital. This new service was implemented subsequent to this analysis, hence the impact of this service change is not captured. There is the opportunity for evaluation of the impact of the new service through our e-Registry with subsequent data extracts. These data provide the opportunity to look at the characteristics of patients and factors affecting their referral for invasive management to support identification of patients who will benefit most from an invasive strategy, and potentially to identify the group of patients for whom referral for angiography is unnecessary, for example those who receive angiography but do not ultimately require PCI. It is currently unknown of this group how many go on to receive cardiac surgery in place of PCI and this will be addressed in the future development of the data set. In addition to this, the ability to record GRACE score for patients who remain in the local hospitals would give further insights into the factors associated with the decision to refer for invasive treatment, or not. The differences observed in the rates of invasive management for male and female patients experiencing NSTEMI warrants further investigation. The statistical analysis suggests that there is no difference in referral rates between men and women, however the crude referral rates suggest, at least a numerical difference. When this is considered in light of a higher proportion of females referred with a GRACE score greater than 140, it would appear that males are more likely to be referred for, and to receive, angiography despite having a larger proportion of low risk scores. This raises questions about the underlying factors influencing the decision to refer for and perform angiography and PCI. The data from the e-Registry presents an opportunity to study other factors associated with gender to better understand the differences in intervention rates and this is an aspect that will be focused on as part of the continuation of the project.

Visibility of this type of information is crucial for local clinicians and decision makers in the NHS to be able to identify focus areas for improved services and outcomes, reduce variation in care and to be able to measure the impact of any changes made. Since the e-Registry is based on linkage using the CHI number, other databases could be linked, such as for drug prescriptions, longer term follow-up in primary care and the ambulance service. The e-Registry has the potential to permit queries at the point-of-care in near-real time following further development of the functionality and creation of an NHS-Focused reporting system.

The ACS e-Registry has its roots in the UK MINAP with some differences. The e-Registry includes variables with definitions that are identical to those that are collected in MINAP. However, the data in the e-Registry can be routinely updated based on executable computer programmes, and no additional manual data recording is needed. Because MINAP involves some *de novo* manual data reporting by individual hospitals, data completeness and accuracy may be qualified, and this is especially the case for patients with NSTEMI or unstable angina. The e-Registry makes use of usual care records without *de novo* data entry, and so, theoretically, patient identification is more complete as recording of diagnosis in the clinical systems is mandatory. Comparison with annual figures for myocardial infarction hospital activity published by Information Services Division (ISD) Scotland^22^ shows that the number of cases of MI identified in the e-Registry is broadly in line with published figures. The methodologies used for each analysis differ. Certain epidemiological methods have been applied to the data from the e-Registry to allow meaningful statistical analysis, such as only considering a patient’s first admission to hospital in the time period studied, therefore it is difficult to draw conclusions on the level of similarity. However, taking these factors into account the number of MI events observed during the study period is consistent with the expected rate for the population served. The benefit of near complete case identification from use of electronic records is contrasted with potential data quality issues. Where data fields in the clinical systems are not mandatory some clinical detail may be less well reported due to poor completion of non-mandatory fields, or poor quality and consistency of data recording in electronic systems.

Three percent of subjects with an ACS had an elective referral. This finding was unexpected and may be explained by how episodes are created. For example, patients with stable symptoms referred on an elective basis experience an ACS while on the waiting list for invasive management, or the referral for an ACS patient is incorrectly categorized as elective by the referring clinician (commonly a trainee doctor). In addition to this, the study period for this analysis started in October 2013. Prior to this date, we do not have any historical data for patients, therefore we may be missing information on prior interactions with the health system which might have been able to clarify what had happened to these patients. This is likely to become less of an issue as new data is added to the e-Registry as patients will have increased history and follow-up.

Despite this limitation, the results of the Data Validation support the conclusion that data extracted from clinical systems to create this e-Registry and the analyses that have been performed on the data set accurately represent what is happening to these patients in the clinical setting. The cause of death for some subjects could not be confirmed using secondary care electronic records, especially for patients who died in the community. This gap points to the need for enhanced communication between primary and secondary care health care systems.

The secondary objective to demonstrate the ability of the pharmaceutical industry to work as a trusted partner to the NHS and academia has been successful. Under the Joint Working Agreement, due to run until April 2019, the skills and resources of all parties have been deployed equally to deliver the ambition of the e-Registry, with the NHS providing clinical leadership, Safe Haven and IT system support, the University providing data science and statistical support and the industry partner providing project management and analytical support. The industry partner has had a unique, enabling role in the development of this e-Registry. Through use of legal and data sharing agreements between all parties, utilizing project-specific anonymized data extracts, which protected individual patient identities and limited access to only appropriate data, the industry partner was able to provide guidance and expertise to the process of managing and analysing the data. The involvement of an industry partner also provided a wider view of the utility and interpretation of the data and brought knowledge of other available data sources for comparison with the e-Registry. For the remainder of the agreement the continued collaboration between the existing partners and inclusion of other stakeholders is viewed as vital to the further development of this work. However, the participation of the industry partner was always intended to be finite; Onward funding and management of the e-Registry will then exclusively rest with the NHS in Scotland.

The future development of the e-Registry should involve other regions in NHS Scotland with the aim of developing a national e-Registry. The current project represents a proof-of-concept ACS e-Registry in one Health Board (GGC), representing approximately 25% of the population in Scotland and could be rapidly upscaled to capture additional areas as the electronic systems and pathways for the other Health Boards in the region are comparable. In addition, linkage with other health-related databases would enable an end-to-end picture of management of patients experiencing ACS. Further to that, by including a broader range of ICD-10 codes for cardiovascular conditions and procedures, the e-Registry could be expanded to include a wider range of cardiovascular patient episodes.

### Limitations

Utilizing data recorded as part of usual care records without *any* additional data capture means that some data completeness and quality issues may exist for non-mandatory or free-text fields. As a result, many of the clinical characteristics collected within the project were only available for patients with a particular diagnosis who followed a particular pathway. The amount of missing data ranged from 0.5% for history of hypertension in STEMI patients intended for invasive management, to over 42% of GRACE scores for all NSTEMI patients (13% in NSTEMI patients referred for angiography). This has shown a need for improved recording of key data elements and this has been fed back to the clinical care teams. In addition, using an automated analysis programme to define episodes of care and categorize the data can present issues when episodes of care are complex or information is incomplete. The assumption that this methodology allows complete capture of patients experiencing ACS relies on accurate recording of diagnoses in the clinical systems. The e-Registry may not have complete clinical records for patients due to the limited data sources included, for example the current lack of data from the primary care and outpatient settings. This issue is being addressed as part of further development. This study does not permit inference on causality, and other interpretations of the data are possible therefore further development and studies are warranted.

## Conclusions

The project has demonstrated that implementation of an e-registry of ACS hospitalizations from existing health records in a complex health care system is possible, without the need for additional data collection or excessive use of NHS resources. It is recommended to look at the electronic systems used to collect these records and address some of the issues surrounding missing data for example making certain fields such as GRACE score mandatory. Also additional guidance for clinicians and administrators should be considered in order to support the consistency and completeness of routinely collected data as part of quality improvement activities to enable the use of these data by the NHS to inform clinical practice and patient care. The data presents further opportunities to study the ACS population in more detail to gain insights into factors affecting clinical pathways and outcomes. The Joint Working Project is an example of how the NHS and pharmaceutical industry can work together to facilitate the delivery of projects that are valuable to the NHS and patients.

## Supplementary material

Supplementary material is available at *European Heart Journal – Quality of Care and Clinical Outcomes* online.

## Supplementary Material

Supplementary Data
