# Supplementary material for: Linking hospital patient records for suspected or established acute coronary syndrome in a complex secondary care system: a proof-of-concept e-registry in National Health Service Scotland
Source: Eur Heart J Qual Care Clin Outcomes. 2018 Feb 16;4(3):155–67. doi: 10.1093/ehjqcco/qcy007 (PMC6030982; doi:10.1093/ehjqcco/qcy007)
Supplement: Supplementary Data [file qcy007_supp.pdf]

Appendix: Supplementary Information

<u>Diagnosis Hierarchy</u>

1. STEMI
2. NSTEMI
3. MI Unspecified
4. Unstable Angina

Supplementary Table 6 Definition of clinical pathways				
Pathway	Pathway Name	Pathway Description	Admission and Discharge Criteria	Additional Criteria
1	Emergency direct to IC	Direct access to IC by emergency services or air ambulance	IC Admission method either 'Direct to Cardiac Centre‘ or 'Air ambulance transfer'	No referral prior to IC admission. Must have valid discharge recorded
2	Via local A&E to IC	Access to IC via emergency services or patient self-presentation at A&E	IC Admission method either 'Ambulance to local A&E‘ or 'Self-referral to local A&E'	No referral prior to IC admission. Must have valid discharge recorded
3	Acute Invasive	Local hospital inpatient admission with referral for direct (bed to bed) transfer to IC for planned invasive treatment	IC Admission method either 'Transfer from ward in local hospital’ or 'Already in cardiac centre'	Local Hospital admission and referral prior to IC admission. Must have valid discharge recorded.
4	Elective Invasive	Local hospital inpatient admission, discharged home with referral to IC for planned invasive treatment	IC Admission method either 'Elective admission‘ or 'Direct to cardiac centre'	Local Hospital admission and referral prior to IC admission. Local hospital discharge must be ‘Home’.
5	Local hospital only	Local hospital inpatient admission with no referral to IC (either died in hospital or were managed conservatively with no planned invasive treatment)	Admission direct to local hospital, discharged home or within local hospital	No referral for further activity after local hospital admission or evidence of IC admission. Must have valid discharge recorded.
6	Elective direct to IC	Direct access to IC for planned invasive treatment with no preceding local hospital inpatient admission	IC Admission method either ‘Elective Admission’ or 'Direct to cardiac centre‘. Discharge method either 'Discharged home’ or 'Inpatient death'	No referral prior to IC admission or evidence of local hospital admission. Must have valid discharge recorded.

Supplementary Table 7. Pathways by final diagnosis

	All (N=2327)	STEMI (N=586)	NSTEMI (N=1068)	Unspecified MI (N=146)	Unstable Angina (N=527)
Pathway in					
Emergency direct to IC	333 (14.3%)	304 (51.9%)	29 (2.7%)	0 (0.0%)	0 (0.0%)
Local A&E to IC	155 (6.7%)	148 (25.3%)	7 (0.7%)	0 (0.0%)	0 (0.0%)
Acute invasive	492 (21.1%)	57 (9.7%)	426 (39.9%)	2 (1.4%)	7 (1.3%)
Elective invasive	208 (8.9%)	5 (0.9%)	198 (18.5%)	0 (0.0%)	5 (0.9%)
Local hospital only	1081 (46.5%)	68 (11.6%)	364 (34.1%)	144 (98.6%)	505 (95.8%)
Elective direct to IC	58 (2.5%)	4 (0.7%)	44 (4.1%)	0 (0.0%)	10 (1.9%)
Pathway out					
Discharged home	1609 (69.1%)	91 (15.5%)	894 (83.7%)	99 (67.8%)	525 (99.6%)
Transferred to local hospital	585 (24.1%)	442 (75.4%)	140 (13.1%)	2 (1.4%)	1 (0.2%)
Death in hospital	133 (5.7%)	53 (9.0%)	34 (3.2%)	45 (30.8%)	1 (0.2%)
Pathway					
Emergency direct to IC					
Discharged home	37 (1.6%)	15 (2.6%)	22 (2.1%)	0 (0.0%)	0 (0.0%)
Transferred to local hospital	279 (12.0%)	273 (46.6%)	6 (0.6%)	0 (0.0%)	0 (0.0%)
Death in hospital	17 (0.7%)	16 (2.7%)	1 (0.1%)	0 (0.0%)	0 (0.0%)
Local A&E to IC					
Discharged home	7 (0.3%)	6 (1.0%)	1 (0.1%)	0 (0.0%)	0 (0.0%)
Transferred to local hospital	135 (5.8%)	130 (22.2%)	5 (0.5%)	0 (0.0%)	0 (0.0%)
Death in hospital	13 (0.6%)	12 (2.1%)	1 (0.1%)	0 (0.0%)	0 (0.0%)
Acute invasive					
Discharged home	316 (13.6%)	15 (2.6%)	295 (27.6%)	0 (0.0%)	6 (1.1%)
Transferred to local hospital	171 (7.4%)	39 (6.7%)	129 (12.1%)	2 (1.4%)	1 (0.2%)
Death in hospital	5 (0.2%)	3 (0.5%)	2 (0.2%)	0 (0.0%)	0 (0.0%)
Elective invasive					
Discharged home	208 (8.9%)	5 (0.9%)	198 (18.5%)	0 (0.0%)	5 (1.0%)
Local hospital only					
Discharged home	983 (42.2%)	46 (7.9%)	334 (31.3%)	99 (67.8%)	504 (95.6%)
Death in hospital	98 (4.2%)	22 (3.8%)	30 (2.8%)	45 (30.8%)	1 (0.2%)
Elective direct to IC					
Discharged home	58 (2.5%)	4 (0.7%)	44 (4.1%)	0 (0.0%)	10 (1.9%)

Data are number (%)

Supplementary Table 8 Service delivery by diagnosis

	All (N=2327)	STEMI (N=586)	NSTEMI (N=1068)	Unspecified MI (N=146)	Unstable Angina (N=527)
Total duration in hospital (days)	6.2 (10.7)	5.5 (8.6)	7.5 (10.9)	11.1 (16.7)	2.9 (8.8)
	3 [2, 6]	3[3, 5]	4 [3, 8]	5 [3, 11]	1 [1, 2]
Local hospital (days)	5.7 (10.7)	4.5 (8.6)	7.0 (11.0)	11.1 (16.7)	2.9 (8.8)
	3 [1, 6]	3 [3, 5]	4 [3, 8]	5 [3, 11]	1 [1, 2]
Intervention Centre (days)	0.6 (1.3)	1.2 (1.6)	0.7 (1.4)	0.0 (0.0)	0.0 (0.2)
	1 [0, 1]	1 [1, 1]	1 [0, 1]	0 [0, 0]	0 [0, 0]
Receipt of coronary angiography					
No	1123 (48.3%)	82 (14.0%)	390 (36.5%)	146 (100.0%)	505 (95.8%)
Yes	1204 (51.7%)	504 (86.0%)	678 (63.5%)	0 (0.0%)	22 (4.2%)
Duration from admission to angiography (days)	6.1 (12.2)	0.5 (2.4)	10.3 (14.6)	- (-)	7.2 (11.8)
Receipt of PCI					
No	1493 (64.2%)	116 (19.8%)	714 (66.9%)	146 (100.0%)	517 (98.1%)
Yes	834 (35.8%)	470 (80.2%)	354 (33.1%)	0 (0.0%)	10 (1.9%)
Duration from admission to PCI (days)	3.6 (9.0)	0.3 (2.1)	8.0 (12.2)	- (-)	3.7 (9.2)
Call to Balloon (min)	110.9 (49.8)	111.0 (49.9)	76.0 (0.0)	-	-
N	326	325	1	0	0
Door to Balloon (min)	39.5 (144.3)	39.1 (144.5)	92.0(128.2)	-	-
N	403	400	3	0	0
Admitted to cardiac ward*					
No	794 (34.1%)	29 (4.9%)	256 (24.0%)	104 (71.2%)	405 (76.9%)
Yes	1533 (65.9%)	557 (95.1%)	812 (76.0%)	42 (28.8%)	122 (23.1%)
Seen by cardiologist*					
No	481 (20.7%)	15 (2.6%)	72 (6.7%)	62 (42.5%)	332 (63.0%)
Yes	1846 (79.3%)	571 (97.4%)	996 (93.3%)	84 (57.5%)	195 (37.0%)

Data are mean (SD) or number (%) or median [IQR].
*Those that went to the invasive centre were automatically classified as yes
